# Soil salinity is the main factor influencing the soil bacterial community assembly process under long-term drip irrigation in Xinjiang, China

**DOI:** 10.3389/fmicb.2023.1291962

**Published:** 2023-10-31

**Authors:** Dongwei Li, Husen Qiu, Guangli Tian, Yulong Zhao, Xinguo Zhou, Shuai He

**Affiliations:** ^1^Farmland Irrigation Research Institute, Chinese Academy of Agricultural Sciences, Xinxiang, Henan, China; ^2^School of Environment and Surveying Engineering, Suzhou University, Suzhou, Anhui, China; ^3^School of Agronomy and Horticulture, Jiangsu Vocational College of Agriculture and Forestry, Jurong, Jiangsu, China; ^4^Northwest Oasis Water-saving Agriculture Key Laboratory, Ministry of Agriculture and Rural Affairs, Xinjiang Academy of Agriculture and Reclamation Science, Shihezi, Xinjiang, China

**Keywords:** long-term drip irrigation, saline-alkali soil, bacterial community, null model, assembly processes

## Abstract

Identifying the potential factors associated with the impact of long-term drip irrigation (DI) on soil ecosystems is essential for responding to the environmental changes induced by extensive application of DI technology in arid regions. Herein, we examined the effects of the length of time that DI lasts in years (N_DI_) on soil bacterial diversity as well as the soil bacterial community assembly process and the factors influencing it. The results showed that long-term DI substantially reduced soil salinity and increased soil bacterial diversity while affecting the soil bacterial community structure distinctly. Null model results showed that the soil bacterial community assembly transitioned from stochastic processes to deterministic processes, as N_DI_ increased. Homogeneous selection, a deterministic process, emerged as the dominant process when N_DI_ exceeded 15 years. Both random forest and structural equation models showed that soil salinity was the primary factor affecting the bacterial community assembly process. In summary, this study suggested that soil bacteria respond differently to long-term DI and depends on the N_DI_, influencing the soil bacterial community assembly process under long-term DI.

## 1. Introduction

Water scarcity and soil salinization are the primary factors influencing the soil environment and sustainable agricultural development in arid regions (Liu X. et al., 2020; Wang Y. H. et al., 2020). Mulched drip irrigation (MDI) has been shown as an effective in conserving water, controlling salinity, and ameliorating soil microenvironments, and is widely used in arid and semiarid regions (Rosegrant et al., 2015; Liu et al., 2017; Thidar et al., 2020; Guan et al., 2022), which combines mulching and DI to uniformly deliver water and nutrients to the root zone through DI tapes, using low-pressure pipes (Beaman and Dillon, 2018; Fan et al., 2018). MDI is a localized irrigation technique (Li et al., 2020) due to the technical characteristics of low-volume and high-frequency irrigation. Cotton planting area has exceeded 2 × 10^6^ ha under MDI as its increasing popularity of DI technology since 1996 in Xinjiang, China (Wang et al., 2019).

MDI leads to huge differences in the soil environment (Phogat et al., 2016; Topak et al., 2016), causing spatial heterogeneity in soil moisture, salinity, nutrients, and temperature (Mmolawa and Or, 2000; Khalil and Baggs, 2005; Hou et al., 2010; Guan et al., 2013). In soil, microbial community composition is greatly influenced by these soil environmental factors (Li et al., 2019; Na et al., 2019; Yang et al., 2020). Relevant studies have shown that MDI can improve enhance microbial activity and abundance (Wang et al., 2008; Neilsen et al., 2014), altering the microbial community structure and composition (Wang et al., 2017, 2021; Tao et al., 2018). In addition, drip irrigation studies have increasingly focused on the effect of short-term regulatory of DI on the soil environment and long-term of DI on soil salinity (Zhang T. B. et al., 2013; Li et al., 2021). However, the effect of long-term application of MDI on the structure and composition of the soil microbial community is not well understood (Merloti et al., 2022). The extensive diversity and heterogeneity of soils are subjected to long-term DI, and the complexity interactions of soil and DI present a great challenge for the comprehensive understanding of the structure and function of microbial communities. Therefore, studying of the evolutionary characteristics of the soil microbial community under long-term DI conditions can facilitate the sustainable development of agricultural ecosystems in arid regions.

Elucidating the underlying mechanism driving the soil microbial community assembly constitutes a major challenge in microbial ecology (Zhou and Ning, 2017). Neutral theory categorizes stochastic patterns (e.g., migration as well as stochastic reproduction and death) and spatial autocorrelation independent of the environment (e.g., diffusion) as stochastic processes (Bell et al., 2005; Vanwonterghem et al., 2014). The mechanisms involved in the establishment of soil microbial communities under disturbances (e.g., land use, irrigation and fertilization, and tillage) using the null model, and infer Community Assembly Mechanisms by Phylogenetic-bin-based null model analysis (iCAMP) have been studied. Previous studies showed that soil environmental properties, such as moisture, nutrients, salinity, and pH, are the primary factors affecting the soil microbial community assembly (Zhang K. et al., 2019; Tang et al., 2021; Lan et al., 2022; Song et al., 2022). Soil microbial community assembly is considered a deterministic process when influenced by factors such as environmental filtering, high salinity (> 1 g/L) reduced the Shannon 1 index of soil bacteria, increased the Chao 1 index of soil fungi, and significantly affected the relative abundance of soil bacterial communities (Liu J. et al., 2020). However, drip-irrigated soils are highly diverse and heterogeneous, and affect the strength of the stochastic and deterministic mechanisms involved in the formation of soil microbial communities (Fournier et al., 2020). In addition, the soil microbial community assembly and its associated mechanisms change dynamically (Dini-Andreote et al., 2015). Temporal dynamics are often related to changes in environmental conditions, making it difficult to elucidate the predominant mechanism underlying the microbial community assembly (Mo et al., 2021). Herein, the characteristics of soil microbial communities under long-term DI conditions in arid regions, particularly the long-term evolution of microbial community assembly, require in-depth investigation.

This study investigated the biogeographic patterns associated with different N_DI_, at four sampling locations in Xinjiang, China, elucidating the soil microbial community composition and assembly. Therefore, we hypotheses that long-term DI affect the soil environment by reducing soil salinity and increasing soil bacterial diversity. The main objectives of this study are (1) to reveal the temporal differences about microbial communities in soils under DI of different time, (2) to describe the ecological assembly process of soil microbial communities under long-term DI conditions, and (3) to identify the primary mechanisms involved in the soil microbial community assembly under long-term DI conditions.

## 2. Materials and methods

### 2.1. Overview of the study area

In July 2021, soil samples were collected from 14 drip-irrigated cotton fields (differing in N_DI_) in 4 farms (85°17′-85°34′E, 44°36′-44°43′N) of the Xinjiang Production and Construction Corps (XPCC) in the Xinjiang Autonomous Region (Supplementary Figure S1). The soils consisted of medium loam with 20.5–24% of physical clay particles (< 0.01 mm in size), a dry volume mass of 1.49–1.61 g/cm^3^, and an average porosity of 41.71%.

Since farms used to be saline wastelands prior to their reclamation in 1997. Following the adoption of DI technology, these farms have been cultivated with cotton crops. Two planting patterns of “one mulch, two tapes, and four rows” and “one mulch, three tapes, and six rows,” were characterized by a mulch width of 120 cm and 210 cm, respectively, and an inter-mulch bare ground width of 60 cm, had been used successively (Ning et al., 2019). Each XPCC farm is irrigated approximately 10 times during the cotton growth period. The irrigation quota ranged from 4,500 to 5,250 m^3^/hm^2^. All the observation sites had been drip-irrigated with well water with a mineral content of 1.3–1.8 g/L, which meets the quality requirement (< 2 g/L) for irrigation water. All the XPCC farms were irrigated and fertilized simultaneously. The applied fertilizer primarily comprised urea (CO(NH_2_)_2_), with a nitrogen content of 46%, and potassium dihydrogen phosphate (KH_2_PO_4_), with a P_2_O_5_ content of 51.5%. Urea and potassium dihydrogen phosphate were applied at rates of 390–585 kg/hm^2^ and 240–330 kg/hm^2^, respectively, throughout the entire growth period.

The fields were categorized by their N_DI_ values, based on the number of years of application of DI technology, in conjunction with the local land management model. Because the different size of all farms according to the actual local situation, three sampling sites with 10, 15, and 20 years of DI application at Point_A were selected, three sampling points with 15, 18, and 25 years of DI application at Point_B were selected, four sampling sites with 8, 10, 15, and 20 years of DI application at Point_C were selected, and four sampling sites with 5, 10, 15, and 20 years of DI application at Point_D were selected, respectively. Supplementary Figure S1 shows the location of the sampling sites.

### 2.2. Sampling and assessment of soil characteristics

All soil samples used in this study were collected in the topsoil (0–20 cm depth) by using multi-point sampling method at the cotton Flower stages. Cutting ring method was used to determine soil volumetric mass at the same time. A total of 84 samples were obtained. The soil samples were classified into three parts, one of which was dried and used for the measurement of physicochemical properties. The second part was stored at 4°C for NH_4_^+^ and NO_3_^−^ and enzyme activity analyses and the other part was stored at −80°C for soil microbial community analyses.

A portion of this subsample was taken as fresh soil, and it was immediately placed in a refrigerator set to 4°C to ascertain the soil indexes (both chemical and physical). We mixed 10 g of the fresh soil with 50 mL of 2 M KCl solution (superior pure GR) in a trigonometric bottle with a capacity of 50 mL and agitated for 15 min (200 rpm) at ambient temperature. After that, the NH_4_^+^ and NO_3_^−^ analyses were conducted on the obtained supernatant utilizing an AA3-HR Continuous Flow Analyzer (Seal Analytical, Germany). A UV–visible spectrophotometer (UV-1200) was utilized to determine the concentration of phosphorus that is readily accessible in the soil. Oven drying technique was used to quantify the soil moisture. Soil organic matter (OM) was determined by the Walkley–Black dichromate oxidation method (Nelson and Sommers, 1982). Total nitrogen (TN) was measured using the Kjeldahl method (FIA Star 5,000 Analyzer; Foss Tecator, Höganäs, Sweden; Bremner, 1996). Total phosphorus (TP) was measured using NaOH digestion and the molybdenum blue colorimetric method (Bao, 2000). Available phosphorus (Olsen-P) was extracted using sodium bicarbonate (Sun et al., 2019). Available potassium was measured by analyzing the filtered extract on an atomic absorption spectrometer (Abd Elwahed et al., 2019). The soil enzyme activities, Alkaline phosphatase (ALP), Polyphenol oxidase (PPO), Urease, and peroxidase (POD), ware estimated by using the method of the β-glucosidase activity (Meena and Rao, 2021). Soil pH was determined based on a soil-to-water ratio of 1:2.5 (w/v). An electrical conductivity meter (DDSJ-308-a) was utilized to test the conductivity of the soil (TDS).

### 2.3. The sequencing of the microbial communities in the soil

With the aid of a FastDNA^®^ SPIN Kit for Soil (QIAGEN, United States), total DNA was isolated from the second subsample of each soil sample. The soil loading weight for DNA extraction is 0.25 g for each sample. The NanoDrop 2000 was adopted to estimate the concentration and purity of the DNA, and the 2% agar-gel electrophoresis method was applied to determine the DNA’s quality. The barcode sequence was included in the process so that sequencing data could be differentiated across samples in 0–20 cm soil layer. Rimers 341F (5′-ACTCCTACGGGAGGCAGCAG-3′) and 806R (5′-GGACTACHVGGGTWTCTAAT-3′) were utilized to amplify the bacterial 16S rRNA gene’s V3-V4 region for each sample (Lu et al., 2013). The procedure for amplifying is as follows: 3 min of pre-denaturation at 95°C; a total of 27 denaturation cycles at 95°C for 30 s each; a 30-s annealing cycle at 55°C; a 30-second extension at 72°C; 10-minute extension at 72°C (PCR instrument: ABI Gene Amp^®^ type 9,700). The overall amount of the amplification system was 20 μL, and it contained the following, which comprised 10 ng of DNA template, 4 μL of 5 × Fast Pfu buffer, 0.4 μL of Fast Pfu polymerase, 0.8 μL of primer (5 μM), and 2 μL of 2.5 mm dNTPs. In addition, 2% agarose gel was utilized to extract PCR products, which were then purified utilizing an Axy Prep DNA Gel Extraction Kit (Axygen Biosciences, Union City, CA, United States), rinsed in Tris–HCl, and finally identified by electrophoresis on a second 2% agarose gel. Subsequent DNA quantification was done with QuantiFluor^™^-ST (Promega, United States). Depending on the sequencing needs and the quantification findings, PCR products were processed to create sequencing libraries. An Illumina NovaSeq 6,000 sequencing system was adopted to sequence the resulting libraries.

Sequencing libraries were filtered to exclude reads with poor quality characteristics such as a low mean Phred score < 20, ambiguous bases, homopolymer runs longer than 6 bases, short sequence lengths (less than 150 bp), and primer mismatches (Bokulich et al., 2013). Additionally, the selected high-quality reads were assigned to samples premised on the unique barcode that was attached to the terminus of the reverse primer. The FLASH algorithm was applied to tag the reads that had an overlap of more than 10 base pairs and did not include any mismatches (Magoc and Salzberg, 2011). With the help of the QIIME v1.9.2 tool, tags that had a similarity of ≥97% were merged into the same operational taxonomic unit (OTU; Caporaso et al., 2010). The default setting was used to select reference sequences for each OTU, and these sequences were then classified into bacterial taxa as per the SILVA database (release 138; Yilmaz et al., 2014). After that, OTU abundance tables of microbial communities were generated and then normalized using standardized labels depending on the sample that included the lowest number of labels.

### 2.4. Statistical analyses

QIIME v1.92 was adopted to compute the Shannon and Chao1 indexes, two measures of the alpha (𝛼) diversity of the soil bacterial community (Kemp and Aller, 2004). Boxplots depicting variations in α diversity indices and soil properties across soil types, time points, and N_DI_ values were generated using R (v4.0.2), and the effects of these parameters were statistically examined via a Tukey’s true significant difference (HSD) test. Principal coordinate analysis (PCoA) and a two-way Adonis test from the “vegan” package in R were conducted to evaluate the variations in the composition of soil microbial population across different time points and N_DI_ values (v4.0.2). By employing the “ggplot2” and “vegan” packages in R (v4.0.2), we performed a redundancy analysis (RDA) to verify the associations of soil microbial populations with soil characteristics.

Stegen et al. (2015) proposed a technique renowned as null modeling to get a deeper comprehension of the mechanisms involved in community assembly. Using the Raup-Crick metric (RC) and the β -Nearest taxon index ( β NTI), we determined the mechanistic basis of the assembly of microbial communities in our samples. We evaluated the relative impacts of heterogeneous and homogeneous selection as the fraction of their comparisons with βNTI > +2 and βNTI < −2, correspondingly. Thereafter, by employing the taxonomic diversity metric RC, which ranges from −1 and 1, we determined that the remaining pairwise comparisons had |βNTI| ≤ 2. Values close to −1 (−0.95 to −1) indicate homogeneous diffusion (i.e., mass effect), those close to 1 (0.95 to 1) indicate diffusion limitation, whereas intermediate values (−0.95 to 0.95) indicate drift. Thus, the remaining fraction of |βNTI| ≤ 2 and |RC| ≤ 0.95 accounted for the percentage of undominated cases.

Random forest analysis was conducted to identify soil properties most strongly linked to bacterial community variation. Linear regressions between actual and predicted PC1 scores for bacterial communities were used to assess the validity of soil properties in evaluating bacterial communities. By measuring the increase in the mean square error when the response variable of each predictor was replaced 999 times at random, we could determine which predictors were the most significant in determining bacterial composition. Finally, the influence of soil characteristics on bacterial community diversity and composition was quantified using structural equation modeling conducted with the “lavven” software package.

## 3. Results

### 3.1. Variations in soil properties

**Figure 1:**
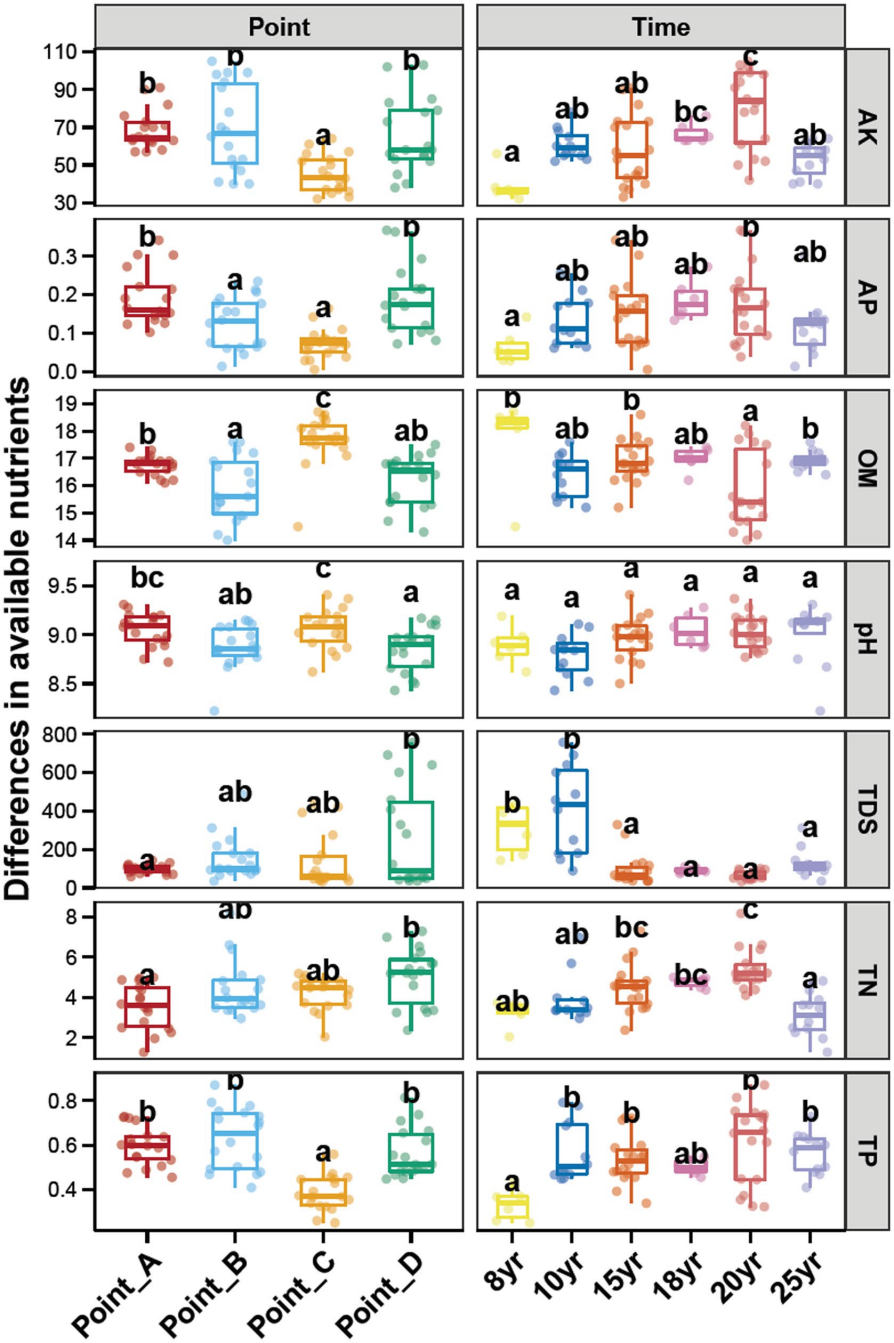
Differences in the contents of soil salt, pH, and nitrate among different points and N_DI_. Different lowercase letters above each box in the same sub-figure represent significant differences between groups (Tukey’s HSD test, *p* < 0.05).

The analysis of the four points selected in this study, from the perspective of main soil environmental factors, is shown in Figure 1. The soils at Point_C had higher organic matter (OM) content than those at the other sampling points (*p* < 0.05), and a higher pH than those at Point_B and Point_D (*p* < 0.05). The available phosphorus (AP) in Point_A and Point_D was higher than that in the other two sampling sites (*p* < 0.05). The soil samples at Point_A had a significantly higher total dissolved solids (TDS) than those at Point_D (*p* < 0.05). The soil pH did not vary considerably with N_DI_. The TDS content of the soils decreased significantly when N_DI_ exceeded 15 years, whereas the TP content increased considerably when N_DI_ exceeded 10 years (*p* < 0.05). The peroxidase (POD) and urease activity of the soils did not vary considerably with the sampling point and N_DI_. The soil that was subjected to DI for 18 years, had the lowest ALP and PPO activity (Supplementary Figure S2).

### 3.2. Change of bacterial communities

**Figure 2:**
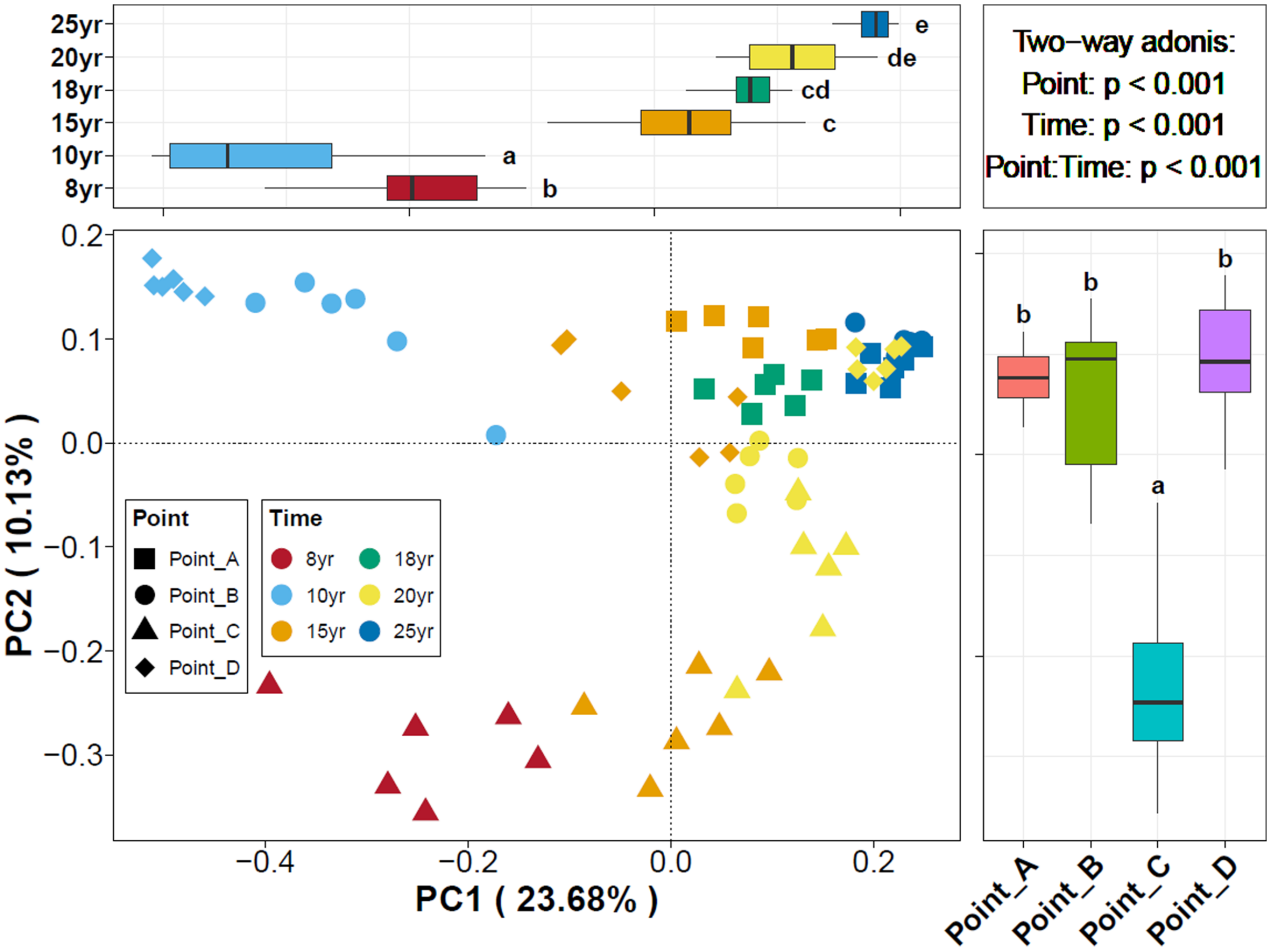
Principal coordinates analysis of soil bacterial communities under different points and N_DI_.

**Figure 3:**
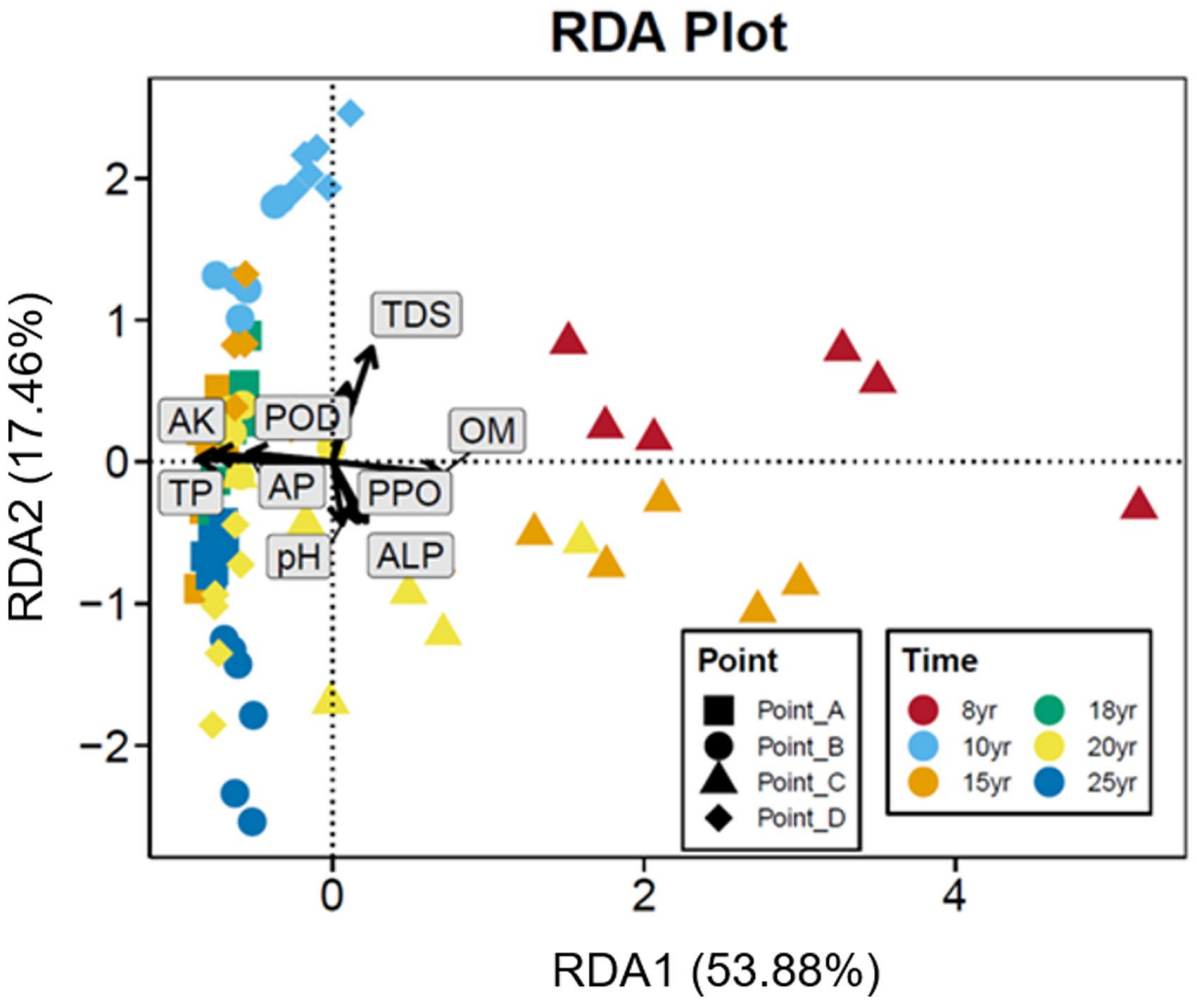
Redundancy analysis showing correlations between bacterial community and environmental variables in different N_DI_ soils.

With N_DI_ increased, the Chao1 index of the soil bacterial communities gradually increased and leveled off after 15 years of DI. In contrast, the Shannon index of the soil bacterial communities began to stabilize at N_DI_ = 10 years. This finding suggests that an increase in N_DI_ considerably increased the diversity and abundance of soil microbes. The principal coordinate analysis (PCoA) revealed that the dissimilarly of soil bacterial community composition changed with time (Figure 2). A correlation analysis of soil properties and microbial community diversity using redundancy analysis (RDA) revealed that RDA1 and RDA2 collectively explained 71.34% of the total variation (Figure 3). TDS (*p* = 0.001), AK (*p* = 0.001), and TP (*p* = 0.001) had a highly significant impact on the differences in soil bacterial communities.

### 3.3. Assembly of soil bacterial communities

**Figure 4:**
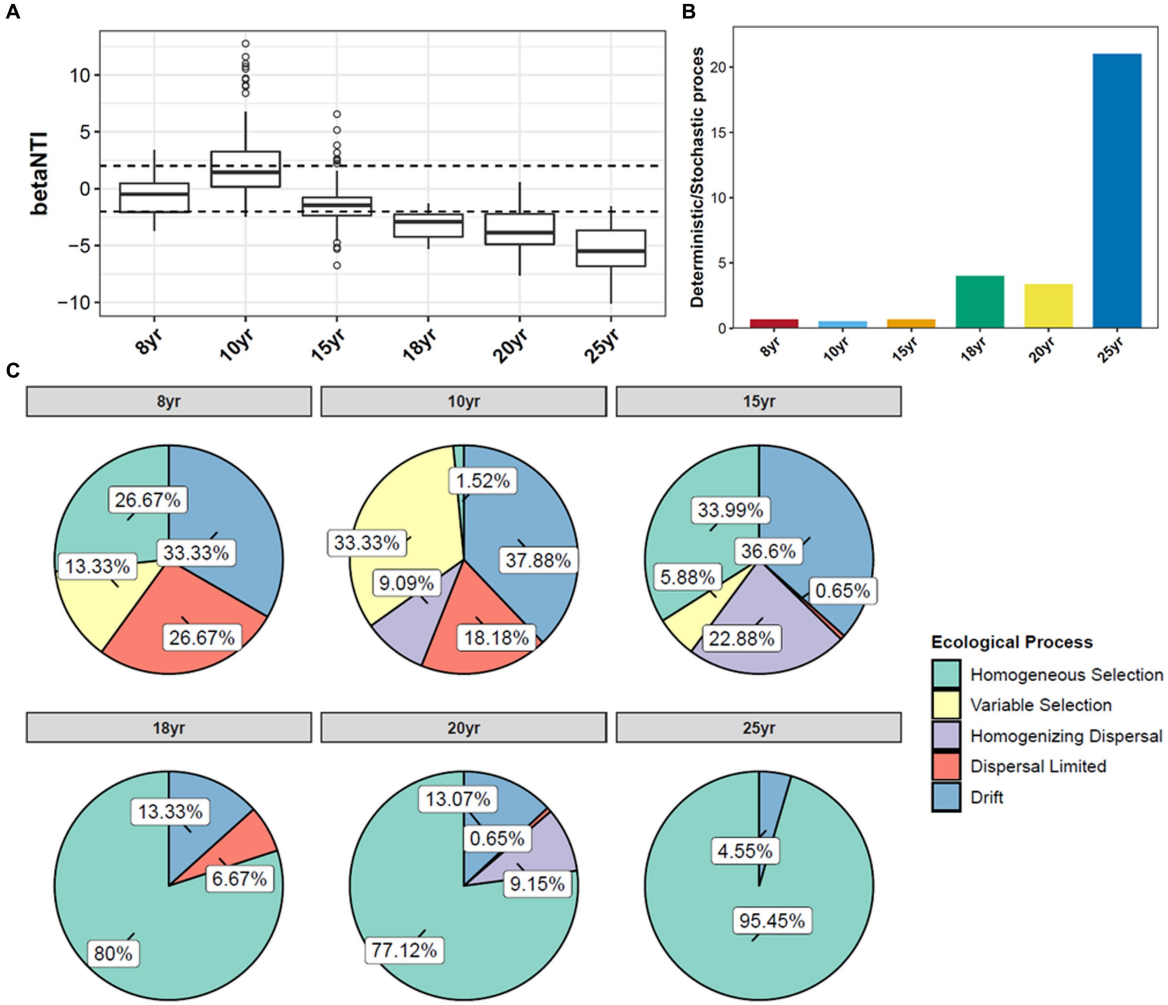
Distribution of the βNTI of soil bacterial **(A)** communities under different N_DI_. **(B)** Deterministic/Stochastic process. **(C)** Null model analysis of the contribution of different factors shaping soil bacterial communities.

The null model analysis revealed a difference between the relative contribution of the deterministic and stochastic processes in the establishment of soil bacterial communities under long-term DI conditions (Figure 4). At N_DI_ < 15 years, stochastic processes played a primary role in shaping soil bacterial communities. At N_DI_ > 15 years, the beta nearest taxon index (betaNTI) value decreased continuously, and the median betaNTI value for each soil sample was lower than −2 (Figure 4A). This suggests that the contribution of deterministic processes in the shaping of soil bacterial communities increased with N_DI_ (Figure 4B). Based on the null model analysis, the deterministic and stochastic processes were further divided into five groups (Figure 4C). The drift of stochastic processes accounted for 33.33–37.88% of the shaping of bacterial communities in soils subjected to DI for less than 15 years. At N_DI_ > 15 years, homogeneous selection, a deterministic process, became the principal ecological process involved in the shaping of soil bacterial communities, accounting for 77.12–95.45% of the process. These findings suggest that soil bacteria under long-term DI conditions are more susceptible to the soil environment.

### 3.4. Factors influencing the assembly of microbial communities

**Figure 5:**
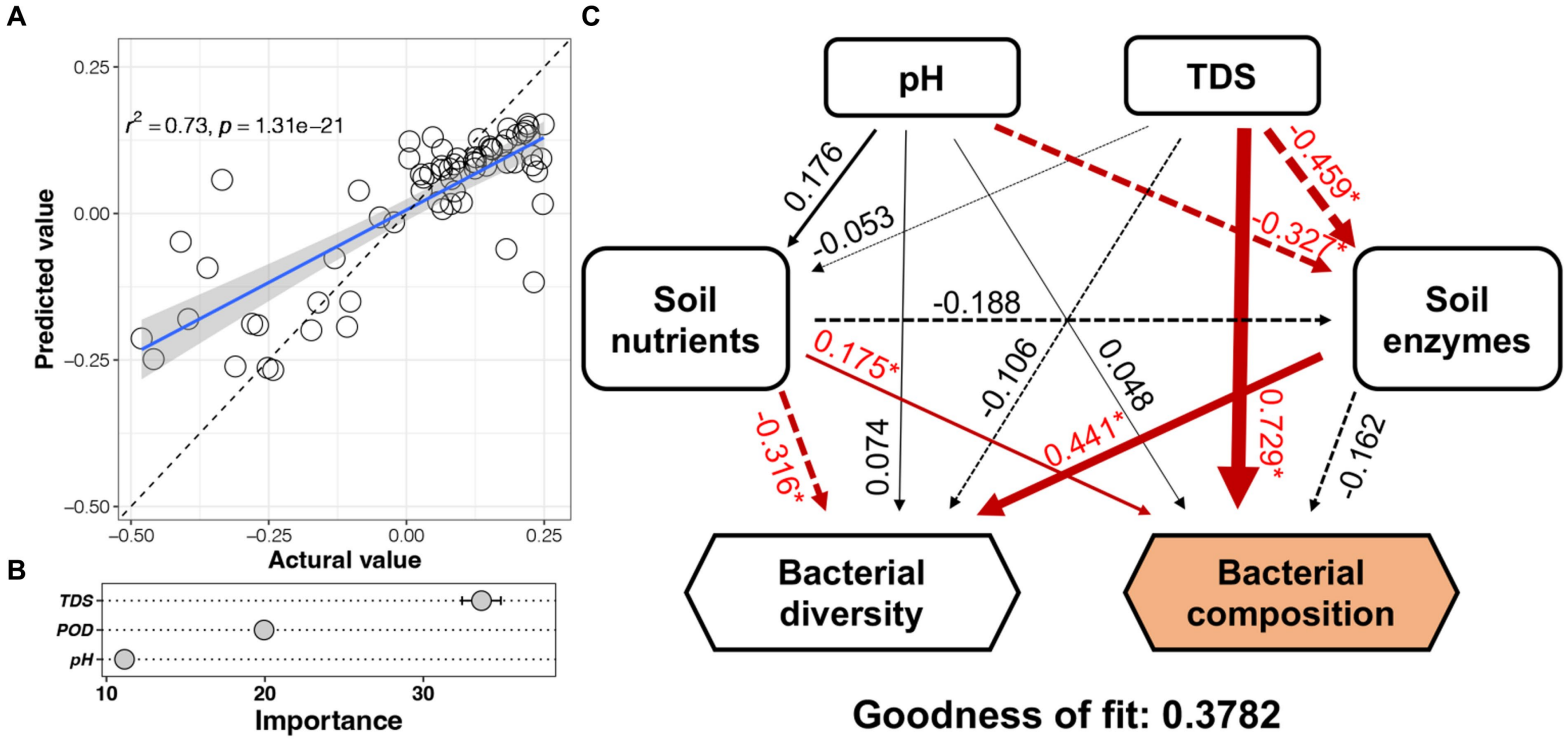
Prediction of soil bacterial community to environmental factors by random forest model and structural equation model. **(A)** The predicted accuracy of the model. **(B)** Important factors. **(C)** Structural equation model. Numbers near to the arrows are coefficients (*p* values). *p < 0.05.

The PC1 values (from the PCoA) of each soil bacterial community were predicted by the random forest model based on soil environmental factors (Figure 5A). The predicted accuracy of this model reached 73% with TDS, POD, and pH as the important factors (Figure 5B). Based on structural equation model (Figure 5C), the soil nutrients exhibited significant negative effects on bacterial diversity and positive effects on bacterial composition, while the enzyme activity exhibited significant negative effects on TDS and pH and positive effects on bacterial diversity, respectively. TDS and soil nutrients had a significant positive impact on the heterogeneity of the bacterial community. Notably, soil salinity had the highest path coefficient (0.729) toward the heterogeneity of the bacterial community. Furthermore, pH and TDS had a significant effect on soil enzyme activity directly, soil enzyme activity impacted the bacterial diversity, that is, the soil environmental factors indirectly affected bacterial diversity.

## 4. Discussion

### 4.1. Effect of long-term drip irrigation on soil salinity and soil bacterial community

MDI plays a crucial role in regulating water and salt transport in irrigated agriculture in arid regions (Ren et al., 2019). MDI is characterized by “shallow irrigation, frequent irrigation, and a small wetting zone (Chen et al., 2009; Palacios-Diaz et al., 2009; Zhang T. B. et al., 2019).” Consequently, MDI is believed to be ineffective in removing the salts from the soil in the crop root zone and is only capable of washing the salts to the edge of the wetting zone during the growth period (Zheng et al., 2009; Liu et al., 2013; Wang et al., 2014). This results in a higher saline content of the soil during the non-growth period than during the growth period and requires washing with large quantities of water during the non-growth period to ensure seedling emergence in the crop in the following year (Sánchez et al., 2015; Bonachela et al., 2018; Tan et al., 2021). Among studies examining the interannual evolution of soil salinity, Zong’s comprehensive study (Zong et al., 2022) demonstrated the efficacy of long-term DI as a viable strategy in reducing soil salinity through irrigation. These findings align with the observations of prior research conducted by Wang et al. (2011), Sun et al. (2012), and Li et al. (2021), although variances in the N_DI_ under scrutiny. The outcomes of the present investigation showed that soil salinity subjected to N_DI_ < 10 years had a considerably higher salinity content than those subjected to N_DI_ > 10 years (Figure 1). Consequently, it becomes evident that the progressive elevation of soil salinity predominantly stems from the regulatory influence of moderated DI, emphasizing the nuanced interplay between irrigation methods and soil salinity dynamics.

**Figure 6:**
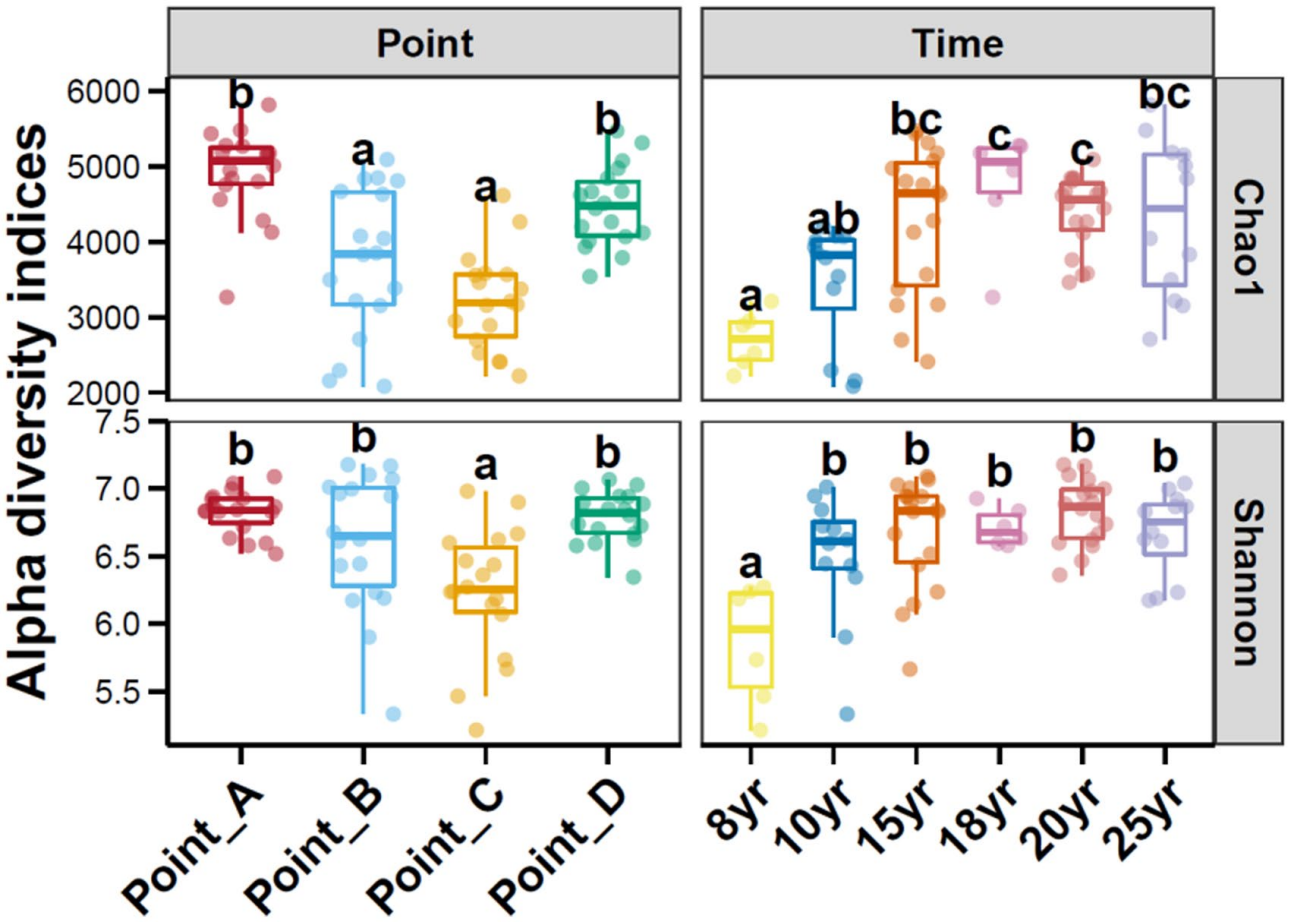
Alpha diversity of soil bacterial communities under different points and N_DI_. Different lowercase letters above each box in the same sub-figure represented the inter-group difference (Tukey’s HSD test, *p* < 0.05).

DI not only creates a suitable water and salt environment for the crop root zone but also affects soil microorganisms while ameliorating the physical and chemical properties of the soil (Liu et al., 2012; Zhang Q. Q. et al., 2013). The results of this study showed that long-term DI significantly increased the alpha diversity of the soil bacteria (Figure 6). This can be primarily attributed to the fact that before the implementation of DI technology, the higher saline content in the soil (Oren, 2011; Rath and Rousk, 2015) increased the extracellular osmotic pressure, forcing microorganism’s incapable of adapting to the osmotic pressure to die or become inactive, leading to a marked decrease in the number, activity, and alpha diversity of soil microorganisms (Pankhurst et al., 2001; Jiao et al., 2006; Ghollarata and Raiesi, 2007). Water and salt regulation through long-term DI aids in increasing the number, diversity, biomass, and metabolic activity of soil microorganisms and the amount of effective soil nutrients with the decrease in salinity gradient (Chen et al., 2017). Similarly, in this study, salinity was the primary factor affecting the variation in the soil microbial community structure (Figure 3). This finding is consistent with that of Lozupone and Knight (2007), Campbell et al. (2013), and Oren (2015).

### 4.2. Relationship of soil salinity and soil bacterial community under long-term drip irrigation

Nonetheless, the extent to which salinity levels constitute the primary determinant influencing the configuration of the microbial community structure remains a subject of ongoing debate. Notably, certain contemporary discoveries postulate an alternative perspective, contending that the intricate framework of the microbial community structure within the soil is distinctly shaped by variables encompassing soil moisture content, pH levels, and nutrient concentrations, supplanting salinity as the paramount factor in this context (Hollister et al., 2010; Zhao et al., 2018; Zhang K. et al., 2019). Soil bacterial diversity emerges as an intricate outcome arising from the intricate interplay of diverse soil factors within a specific environment. The persistent perturbation or disruption of the soil fabric, denoted as prolonged and sustained disturbance (DI), wields the potential to induce alterations in fundamental soil attributes, thereby perturbing the innate equilibrium of the soil ecosystem. Notably, the continuum of prolonged DI can exert a transformative influence on the dynamic process of soil salinity, thereby orchestrating a cascade of effects on the intricate tapestry of soil biodiversity (Pino et al., 2019). This intricate nexus linking prolonged disturbance, the intricate modulation of soil salinity, and the multifaceted landscape of soil bacterial diversity encapsulates a nuanced interrelationship, resonating with consequential ramifications on the others. The results of this study showed that soil salinity was the main cause of the changes in the soil microbial community structure, and AK and TP content also affected the soil microbial community structure significantly (*p* = 0.001; Figure 3), which is consistent with the results obtained by Zhao et al. (2018) and Hollister et al. (2010). These results suggest that salinity may be responsible for the changes in soil bacterial diversity and community structure induced by long-term DI.

Stochastic and deterministic processes play different roles in the soil microbial community assembly, depending on environmental conditions, the successional stage, and the type and level of disturbance (Liu J. et al., 2020). The results of this study showed that stochastic processes played a dominant role at N_DI_ < 15 years, whereas deterministic processes played an increasingly critical role as N_DI_ increased (Figure 4C). This suggests that stochastic and deterministic processes simultaneously play a role in the succession of soil bacterial communities under long-term DI conditions, and that their significance changes as N_DI_ increases, which is consistent with the results of Ofiteru et al. (2010), Caruso et al. (2011), Zhang et al. (2021), and Zheng et al. (2021) that stochastic and deterministic processes are jointly involved in the microbial community assembly. This may be attributed to the fact that when applied to saline-alkaline soil, MDI destroys the original fixed soil environment, resulting in corresponding changes in the physical and chemical properties of the soil. Such change can stimulate species migration process and stochastic colonization processes (Jia et al., 2018; Kou et al., 2020). Different soil environments contain different microbial communities until the soil ecosystem reaches a new equilibrium (Galand et al., 2016; Zhou and Ning, 2017). Analysis of the results for the soil environmental factors (Figure 1) and soil microbial diversity (Figures 2, 6) revealed that this equilibrium point may occur approximately at an N_DI_ of 15 years. Marked changes in environmental factors may induce deterministic processes, which may be the cause of the increase in deterministic processes at N_DI_ > 15 years. Moreover, homogeneous selection leads to a more similar microbial community structure, contributing to a more stable soil bacterial community (Li et al., 2019). In this study, a random forest model and a structural equation model were used to identify soil environmental factors affecting soil bacterial communities (Figure 5), and to analyze the contribution of the soil environmental factors in the soil bacterial community assembly (Figure 4), respectively. The results of both the analyses showed that soil salinity was the primary factor influencing the soil bacterial community assembly under long-term DI conditions, which is consistent with the findings of the study by Zhang K. et al. (2019) and Li et al. (2022) that salinity is a major factor affecting the soil bacterial community assembly. This study suggests that the transformation of long-term DI occurs through salinity regulation processes, encompassing stochastic, selection, and stabilization processes (Dini-Andreote et al., 2015).

## 5. Conclusion

The results showed that long-term DI substantially reduced soil salinity and considerably increased soil bacterial diversity at N_DI_ > 10 years while affecting the soil bacterial community structure distinctly. The null model results showed that homogeneous selection was the dominant process at N_DI_ > 15 years. Both random forest and structural equation models showed that soil salinity was the primary factor impacting the bacterial community assembly process. These results provide new insights into the sustainable productivity of agricultural ecosystems in arid regions and can further facilitate the sustainable development of drip-irrigated agricultural ecosystems in arid regions.

## Data availability statement

The raw data supporting the conclusions of this article will be made available by the authors, without undue reservation.

## Author contributions

DL: Conceptualization, Investigation, Writing – original draft. HQ: Data curation, Writing – review & editing. GT: Formal analysis, Methodology, Writing – review & editing. YZ: Writing – review & editing. XZ: Investigation, Writing – review & editing. SH: Formal analysis, Writing – review & editing.
