# Supplementary material for: Soil salinity is the main factor influencing the soil bacterial community assembly process under long-term drip irrigation in Xinjiang, China
Source: Front Microbiol. 2023 Oct 31;14:1291962. doi: 10.3389/fmicb.2023.1291962 (PMC10644797; doi:10.3389/fmicb.2023.1291962)
Supplement: Supplementary file 1 [file Data_Sheet_1.pdf]

Figure S1 Geographical Location of the Study Area.

Figure S2 Differences in the soil enzymatic activity among different points. Different lowercase letters above each box in the same sub-figure represent significant differences between groups (Tukey's HSD test, $p < 0.05$).

Figure S1

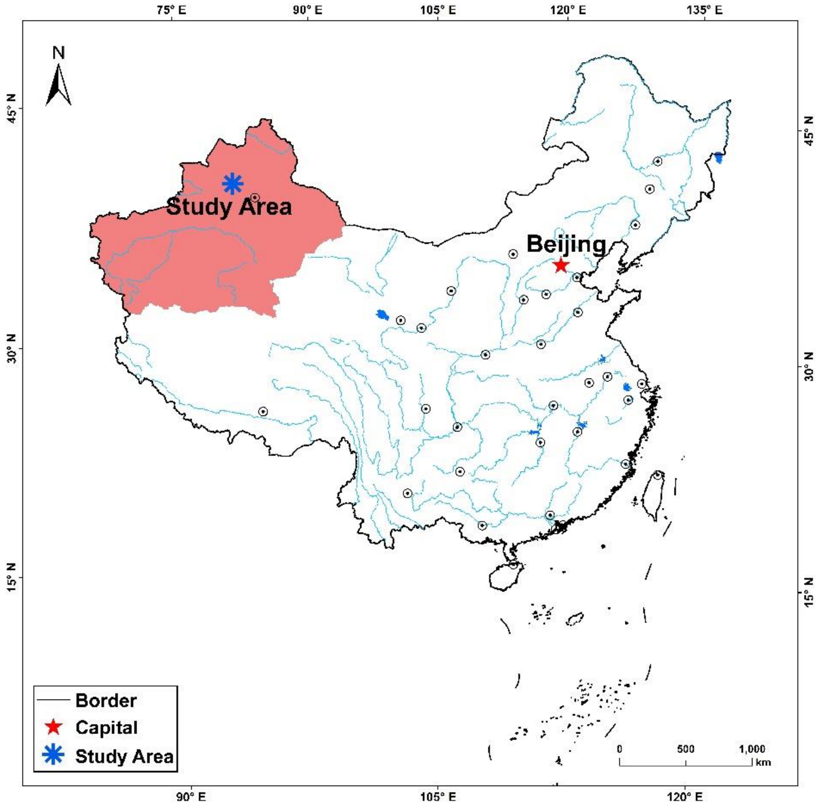
75° E
90° E
105° E
120° E
135° E
45° N
30° N
15° N
N
Study Area
Beijing
Border
Capital
Study Area
0
500
1,000
km

Figure S2

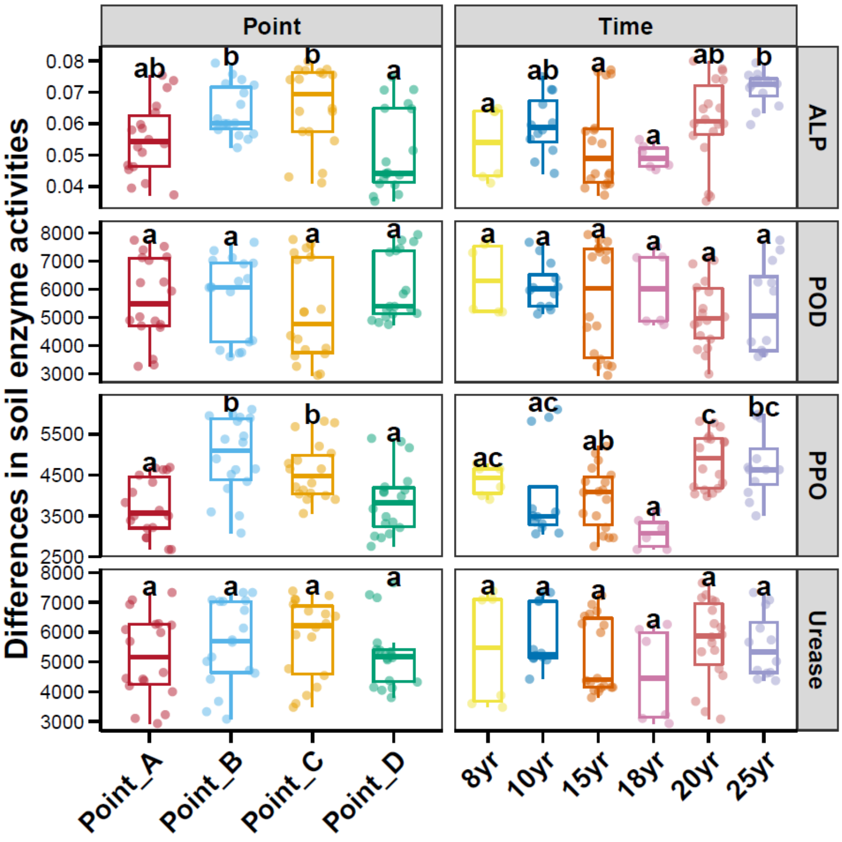

Point
Time
Differences in soil enzyme activities
ALP
POD
PPO
Urease
0.08
0.07
0.06
0.05
0.04
8000
7000
6000
5000
4000
3000
5500
4500
3500
2500
Point_A
Point_B
Point_C
Point_D
8yr
10yr
15yr
18yr
20yr
25yr
